# Clinical decision making: validation of the nursing anxiety and self-confidence with clinical decision making scale (NASC-CDM ©) into Spanish and comparative cross-sectional study in nursing students

**DOI:** 10.1186/s12912-024-01917-w

**Published:** 2024-04-24

**Authors:** Daniel Medel, Tania Cemeli, Krista White, Williams Contreras-Higuera, Maria Jimenez Herrera, Alba Torné-Ruiz, Aïda Bonet, Judith Roca

**Affiliations:** 1 Department of Nursing and Physiotherapy, University of Lleida, 2 Montserrat Roig, St., 25198 Lleida, Spain; 2 School of Nursing, Georgetown University, Washington, DC USA; 3 Open University of Catalonia (UOC), Barcelona, Spain; 4 Department of Nursing, University Rovira Virgili, Tarragona, Spain; 5 Xarxa Assistencial Universitària de Manresa, Hospital Fundació Althaia, Manresa, Spain; 6 Health Education, Nursing, Sustainability and Innovation Research Group (GREISI), Lleida, Spain

**Keywords:** Anxiety, Clinical decision-making, Nursing students, Self confidence, Reliability, Validity

## Abstract

**Background:**

Decision making is a pivotal component of nursing education worldwide. This study aimed to accomplish objectives: (1) Cross-cultural adaptation and psychometric validation of the Nursing Anxiety and Self-Confidence with Clinical Decision Making (NASC-CDM©) scale from English to Spanish; (2) Comparison of nursing student groups by academic years; and (3) Analysis of the impact of work experience on decision making.

**Methods:**

Cross-sectional comparative study. A convenience sample comprising 301 nursing students was included. Cultural adaptation and validation involved a rigorous process encompassing translation, back-translation, expert consultation, pilot testing, and psychometric evaluation of reliability and statistical validity. The NASC-CDM© scale consists of two subscales: self-confidence and anxiety, and 3 dimensions: D1 (Using resources to gather information and listening fully), D2 (Using information to see the big picture), and D3 (Knowing and acting). To assess variations in self-confidence and anxiety among students, the study employed the following tests: Analysis of Variance tests, homogeneity of variance, and Levene’s correction with Tukey’s post hoc analysis.

**Results:**

Validation showed high internal consistency reliability for both scales: Cronbach’s α = 0.920 and Guttman’s λ2 = 0.923 (M = 111.32, SD = 17.07) for self-confidence, and α = 0.940 and λ2 = 0.942 (M = 80.44, SD = 21.67) for anxiety; and comparative fit index (CFI) of: 0.981 for self-confidence and 0.997 for anxiety. The results revealed a significant and gradual increase in students’ self-confidence (*p* =.049) as they progressed through the courses, particularly in D2 and D3. Conversely, anxiety was high in the 1st year (M = 81.71, SD = 18.90) and increased in the 3rd year (M = 86.32, SD = 26.38), and significantly decreased only in D3. Work experience positively influenced self-confidence in D2 and D3 but had no effect on anxiety.

**Conclusion:**

The Spanish version (NASC-CDM-S©) was confirmed as a valid, sensitive, and reliable instrument, maintaining structural equivalence with the original English version. While the students’ self-confidence increased throughout their training, their levels of anxiety varied. Nevertheless, these findings underscored shortcomings in assessing and identifying patient problems.

## Background

Decision making in nursing is a critical process that all nurses around the world use in their daily practice, involving the assessment of information, the identification of health issues, the establishment of care objectives, and the selection of appropriate interventions to address the patient’s health problems [1, 2]. Nursing professionals must effectively apply their knowledge, skills, and clinical judgment to ensure the delivery of safe and high-quality care within the context of complex and ever-evolving situations [3]. For nearly 25 years, clinical decision-making has been highlighted as one of the key aspects of nursing practice [2, 4].

Decision making in nursing does not follow a linear relationship that culminates in the decision made; instead, it has a circular nature that repeats through data collection, alternative selection, reasoning, synthesis, and testing [5]. Expert nurses, moreover, possess the ability to discern patterns and trends within clinical situations, providing them with a general overview of patient issues and facilitating decision making [6]. In this iterative and dynamic process, a solid knowledge base, clinical experience, reliable information, and a supportive environment are crucial pillars underpinning clinical decisions [7]. Therefore, nursing students, during their educational journey, require the support of others in decision making [4] and adequate training that optimizes their learning opportunities [8]. Clinical decision-making forms the cornerstone of professional nursing practice [9].

The process of decision making regarding patient care integrates theoretical knowledge with hands-on experience [10]. This practical experience has been instrumental in augmenting analytical skills, intuition, and cognitive strategies essential for determining sound judgment and decision-making in complex situations [11]. Although students’ clinical experience is limited, some of them work as nursing assistants or in support roles. This profile of nursing student is quite common [12]. Hence, prior work experience in healthcare should be considered in nursing students.

Additionally, it has been suggested that emotional factors, such as heightened levels of anxiety and low self-confidence, may influence clinical decision-making processes [13]. The Nursing Anxiety and Self-Confidence with Clinical Decision Making (NASC-CDM©) scale is used to make a self-report of how they feel about students’ self-confidence and anxiety levels during clinical decision-making [14] On one hand, nursing students frequently grapple with elevated stress and anxiety, which adversely affect their learning process [15]. Conversely, self-confidence is defined as a person’s self-recognition of their abilities and capacity to recognize and manage their emotions [16]. Self-confidence can foster well-being by strengthening positive emotions among nursing students [17]. In this regard, one of the leading authors in the study of self-confidence is Albert Bandura (1977) [18]. He employs the term self-efficacy to describe the belief that one holds in being capable of successfully performing a specific task to achieve a given outcome. Consequently, it can be considered a situationally specific self-confidence [19]; however, these terms are related to potential emotional barriers in decision making [20].

In line with the aforementioned, and as a rationale for this study, it should be noted that the NASC-CDM© scale offers significant contributions. Firstly, it highlights the ability to address self-reported levels of self-confidence and anxiety, both independently and interrelatedly, as these two are two distinct constructs with relevant effects on clinical decision making. This separation allows for a more comprehensive and precise understanding of the context [21]. Secondly, it is worth noting that the scale can be administered to both students and professionals [22]. The results obtained through this scale enable the identification of areas in which students need improvement and provide nursing educators the opportunity to develop strategies to strengthen students’ clinical decision-making skills [14].

The absence of a validated Spanish version of the Nursing Anxiety and Self-Confidence with Clinical Decision Making (NASC-CDM©) scale poses a significant challenge for researchers and educators. This limitation hinders the accurate assessment of self-confidence and anxiety levels among Spanish-speaking nursing students and professionals in both clinical decision-making both academic and healthcare settings. In heath research, the availability of reliable measurement tools is crucial to ensure accuracy and comparability across cultural and linguistic contexts [23]. Moreover, it is noteworthy that the NASC-CDM© scale is not only accessible in English [14] but also in other languages such as Turkish [24] and Korean [22], Therefore, its availability in Spanish presents numerous opportunities for cross-cultural comparisons in academic and healthcare settings, as well as between academic and clinical researchers.

Hence, this study aims to address two deficits in the Spanish context: first, to validate the NASC-CDM© scale in Spanish, and second, to employ it to assess self-confidence and anxiety levels in decision making among nursing students by academic year and the influence of prior work experience. By achieving these objectives, the study seeks to provide educators with essential insights to enhance the teaching and learning process in both academic and environments. Additionally, it aims to offer support students in enhancing their decision-making skills, ultimately fostering the development of proficient healthcare professionals capable of delivering care. Therefore, this study was designed to achieve three primary objectives: (1) To perform a cross-cultural and psychometric validation of the Nursing Anxiety and Self-Confidence scale with the Clinical Decision Making (NASC-CDM©) from English to Spanish Nursing Anxiety and Self-Confidence with Clinical Decision Making– Spanish (NASC-CDM-S©) scale.; (2) To compare groups of nursing students from their first to fourth academic year in terms of anxiety and self-confidence in their decision-making processes; and (3) To Investigate the potential impact of the participants’ work experience on their decision-making abilities. Hence, concerning objectives 2 and 3, the following hypothesis was posited: participants in higher academic years and participants with work experience have higher levels of self-confidence and lower levels of anxiety in their decision-making processes.

## Methods

### Design

This study adopted a quantitative cross-sectional and analytical approach.

### Setting and sampling

The study population comprised nursing students from the Faculty of Nursing and Physiotherapy, University of Lleida (Spain). The nursing degree program in Spain consists of 240 European Credit Transfer System (ECTS) credits, approximately equivalent to 6000 h, distributed across 4 academic years (60 ECTS per year, totaling 1500 h per year). One ECTS credit corresponds to 25–30 study hours (Royal Decree 1125/2003). The first year primarily focuses on theoretical training in basic sciences, with more specific nursing sciences covered in higher years. Clinical practices gradually increase, with the fourth year being predominantly practical (1st year 6 ECTS, 2nd year 12 ECTS, 3rd year 24 ECTS, and 4th year 39 ECTS).

A convenience sample of 301 participants was used, representing a non-probability sampling method [25]. The sample size aligns with the recommended person-item ratio, with a minimum of 10 subjects per item for general psychometric approaches and 300–500 for confirmatory factor analysis (CFA) or conducting propriety analysis [23]. The NASC-CDM© scale contains 27 items. Inclusion criteria were nursing students from all four academic years who were willing to participate, and no exclusion criteria were specified. Participants received no compensation, and their participation was voluntary.

### Instrument and variables

The original version of the NASC-CDM© tool was developed by White [14, 21]. The use of this tool for the study was authorized in May 2022 through email communication with the instrument’s creator.

Regarding the original instrument, it is noteworthy that it was validated through an exploratory factor analysis (EFA) with 545 pre-licensure nursing students in the United States. The analysis revealed moderate convergent validity and significant correlations between the self-confidence and anxiety variables that constitute two separate sub-scales within the same instrument. The instrument achieved a Cronbach’s α of 0.98 for self-confidence and 0.94 for anxiety [14, 21]. This instrument comprises 27 items and uses a 6-point Likert scale for responses (1 = Not at all; 2 = Only a little; 3 = Somewhat; 4 = Mostly; 5 = Almost completely; 6 = Completely). Scores range from 27 to 162 points. The EFA results confirmed a scale with three dimensions (D1, D2, and D3):

1. D1 (Using resources to gather information and listening fully) includes statements about recognizing clues or issues and assessing their clinical significance. This dimension comprises 13 items, with a minimum score of 13 and a maximum of 78.
2. D2 (Using information to see the big picture) includes statements about determining the patient’s primary problem. This dimension contains 7 items, with a minimum score of 7 and a maximum of 42.
3. D3 (Knowing and acting) includes statements about performing interventions to address the patient’s problem. This dimension consists of 7 items, with a minimum score of 7 and a maximum of 42.

Based on the original tool, the questionnaire used in this study consisted of two parts. It included the following variables: (a) sociodemographic data such as age (numeric), gender (male, female, non-binary), academic year (1st, 2nd, 3rd, 4th), university entrance pathway (secondary school, training courses, other university degrees, over 25–45 years old), and participants’ work experience in healthcare (Yes or No); and (b) 27 paired statements about students’ perceptions of their level of self-confidence and anxiety (dependent variable) in decision making as per the translated NASC-CDM©. Regarding work experience, it should be noted that some nursing students work in healthcare facilities as nursing assistants or in support roles during their nursing studies.

### Instrument validation

The tool presented by White [14] underwent translation and adaptation, following the guidance provided by Sousa & Rojjanasrirat [23] and Kalfoss [26]. In the forward-translation (English to Spanish) and back-translation phases, two independent bilingual translators participated, who were not part of the research team and who usually work with health-related translations. The back-translated version of the scale was reviewed and approved by the tool’s creator (Dr. White). These steps ensured content validity.

In the expert panel phase, 5 expert nurse educators from our university who were not part of the research team, with a doctoral degree and more than 5 years of teaching experience, assessed content relevancy. The scale proposed by Sousa & Rojjanasrirat [23] (1 = not relevant, 2 = unable to assess relevance, 3 = relevant but needs minor alteration, 4 = very relevant and succinct), along with the Kappa index were used to assess agreement. The educators rated the 27 items between 3 and 4. The concordance analysis yielded a score of 0.850, which, as per Landis & Koch [27], is considered nearly perfect. Only some expressions were modified for better cultural adaptation while retaining the original meaning of the statements. Finally, a pilot test was conducted during the pre-testing phase, involving 20 students, to assess comprehension and completion time. The students encountered no comprehension difficulties, and the average response time was 13 min. Therefore, it was concluded that the questionnaire was feasible in terms of time required taken and clarity of the questions/answers [28].

This validation process concludes with the psychometric testing of the prefinal version of the translated instrument. During this phase, the psychometric properties are established using a sample from the target population, in this case, nursing students [23]. The psychometric characteristics examined include: (1) the reliability of internal consistency (Cronbach’s Alpha coefficient (α) and Guttman split-half coefficients (λ2); (2) criterion validity, where the concurrent validity of the new version of the instrument was assessed against the original version via confirmatory factor analysis (CFA), and (3) for construct and structural validity, exploratory factor analysis (EFA) and CFA were conducted to demonstrate the discriminant validity of the instrument by comparing groups within the sample.

### Data collection

Data collection took place between May 2022 and June 2023. The lead researcher in a classroom administered the questionnaire in a paper format. Response times ranged from 10 to 15 min.

### Data analysis

A descriptive statistical analysis of the participants’ study variables was conducted. Reliability was determined using Cronbach’s Alpha coefficient (α) and Guttman split-half coefficients (λ2) for both sub-scales (self-confidence and anxiety) and their respective dimensions (D1, D2, D3). Cronbach’s provides a measure of item internal consistency, while Guttman split-half coefficient assesses the extent to which observed response patterns align with those expected from a perfect scale [29]. Item correspondence was reviewed by repeating the exploratory factor analysis (EFA) using the extraction and rotation methods outlined by the tool’s creator [14, 21]. Factor validity was confirmed through confirmatory factor analysis (CFA), where a value ≥ 0.9 of the fit indices (comparative fit index (CFI), Tucker-Lewis Index (TLI), Bentler-Bonett Non-normed Fit Index (NNFI), and Bollen’s Incremental Fit Index (IFI) indicate reasonable fit [30]. The root mean square error of approximation (RMSEA) and the unweighted least square (ULS) estimator was used Likert ordinal data [31]. Sample adequacy was also reviewed using Kaiser-Meyer-Olkin (KMO), Bartlett’s sphericity test, and average variance extracted (AVE).

Normality tests for self-confidence and anxiety data distribution (*N* = 301) were performed using Kolmogorov-Smirnov test (K-S = 0.043 and 0.41; *p* >.05) and multivariable normality (Shapiro-Wilk = 0.993 and 994; *p* >.05). The results indicated that all dimensions followed a normal distribution. Consequently, parametric tests such as Pearson’s correlation coefficient (r) and group comparison tests (t-Student) were employed. To analyze differences in self-confidence and anxiety among students by academic year (1st, 2nd, 3rd, 4th), the following tests were conducted: analysis of variance (ANOVA) tests, homogeneity of variance tests, and Levene’s test applying Tukey’s post hoc correction to *p*-values for combined groups correction for combined groups. Effect sizes were determined using Cohen’s d for t-student tests and eta-squared (η²) for ANOVA tests.

Data were analyzed using IBM SPSS Statistics 24 and JASP 0.18.1. A significance level was set at *p* <.05 for all analyses.

## Results

The results are presented in 4 sections: (1) Descriptive data of the participants, (2) Psychometric validation study of the NASC-CDM© questionnaire in Spanish (NASC-CDM-S©), (3) Comparative analysis of self-confidence and anxiety in decision making by academic year, and (4) The impact of students’ work experience on their decision-making processes.

### Descriptive data of the participants

The nursing study involved 301 participants, mostly women who entered through high school. The sample comprised students from the 1st year of the degree (28.57%, with an average age of 20.43 years), 2nd year (38.54%, with an average age of 21.10 years), 3rd year (3.29%, with an average age of 23.90 years), and 4th year (19.60%, with an average age of 22.92 years). Nearly 2/3 of the participants entered the nursing program from secondary school, and just over 50% had work experience in healthcare. See Table 1 for Sample Characteristics.

**Table 1:** Characteristics of the sample: number (N) and frequencies (%)

Variables		*N* = 301	%
Age*		21.60	3.98 (SD)
Gender	Male	48	15.95%
	Female	252	83.72%
	Nonbinary	1	0.33%
Pathway to university	Secondary school	204	67.77%
	Training courses	80	26.58%
	Other university degrees	7	2.33%
	Over 25–45 years old	10	3.32%
Nursing degree year	1^r^	86	28.57%
	2nd	116	38.54%
	3rd	40	13.29%
	4th	59	19.60%
Work experience	Yes	169	56.15%
	No	132	43.85%

* Mean and standard deviation (SD)

### Psychometric validation study of the NASC-CDM© questionnaire in Spanish

The set of items showed high internal consistency reliability in both sub-scales. In self-confidence, Cronbach’s α = 0.920, and Guttman’s λ2 = 0.923 (M = 111.32, SD = 17.07) and in anxiety the values were α = 0.940 and λ2 = 0.942 (M = 80.44, SD = 21.67). The KMO adequacy measure was 0.921 for self-confidence and 0.946 for anxiety, and Bartlett’s sphericity was highly significant, resulting in a *p*-value not exceeding 0.05, indicating a significantly different item correlation matrix (self-confidence χ2 = 4250.632, *p* <.001; anxiety χ2 = 5612.051, *p* <.001). In addition, the average variance extracted (AVE) index exceeded 0.50, confirming the suitability of the original variables in both sub-scales for structure detection.

To confirm the validity of the factors, agreement of item alignment with the dimensions of the original tool was first examined through EFA (factor loading > 0.4), followed by a confirmatory analysis of the entire scale using CFA. Repeating the EFA, as conducted by White (2011) using alpha factoring extraction and Promax rotation with 3 factors (no eigenvalue), the total variance explained in both scales was 48.30% in self-confidence and 55.30% in anxiety, with an average of 51.80%. The agreement between the items in the resulting factor structure matrix from the EFA and the original matrix were very similar for the anxiety sub-scale (89.90%) but only moderately similar for the self-confidence sub-scale (59.30%), where items did not fall within the same dimensions.

Given the low result, a CFA was conducted based on the dimensions proposed by White (2011). The goodness-of-fit indicators of the model were: (CFI, IFI = 0.981, TLI, NNFI = 0.979, and RMSEA = 0.052) for self-confidence and (CFI, TLI, NNFI, IFI = 0.997 and RMSEA = 0.024) for anxiety. This indicates that the three-factor model retains the description with the original items.

Table 2 shows the estimated factor loadings by dimension and item, illustrating the robust composition of the dimensions with no item elimination. Although items Q5, Q27 and Q11 had factor loadings below 0.60, their KMO values were ≥ 0.80, indicating adequate sampling.

**Table 2:** Confirmatory factor analysis by dimensions and items (*Spanish version NASC-CDM-S©)

			Self-Confidence				Anxiety			
Dimension (D)	Q	Item	load factor	KMO	Item r	M ± SD	load factor	KMO	Item r	M ± SD
**D1** Using resources to collect information and listening carefully			α = 0.888 λ2 = 0.890				α = 0.903 λ2 = 0.904			
	8	Discuss your findings with the instructor.	0.63	0.880	0.56	4.72 ± 1.08	0.70	0.903	0.62	2.56 ± 1.32
	9	Active listening in the interview.	0.66	0.907	0.64	4.89 ± 0.98	0.79	0.932	0.73	2.34 ± 1.21
	10	Assess non-verbal language.	0.62	0.890	0.56	4.48 ± 1.08	0.71	0.925	0.63	2.48 ± 1.16
	11	Review protocols or clinical literature.	0.57	0.909	0.51	4.40 ± 1.16	0.63	0.946	0.31	2.76 ± 2.14
	12	Take into account the information of your partner or family.	0.70	0.932	0.62	4.52 ± 1.02	0.75	0.938	0.68	2.44 ± 1.10
	16	Shift change report information.	0.62	0.946	0.52	4.42 ± 1.00	0.76	0.973	0.65	2.66 ± 1.18
	18	Ask questions for additional information.	0.68	0.921	0.57	4.58 ± 1.12	0.75	0.953	0.67	2.56 ± 1.26
	19	Assess coincidences between physical examination and non-verbal signals.	0.69	0.954	0.53	4.17 ± 1.04	0.74	0.960	0.66	2.82 ± 1.16
	22	Discuss with the instructor the interventions that I consider.	0.64	0.861	0.57	4.76 ± 1.08	0.75	0.909	0.66	2.51 ± 1.31
	23	Think about other possible causes of the problem.	0.67	0.922	0.62	4.50 ± 1.12	0.78	0.959	0.72	2.56 ± 1.24
	24	Ask third parties for information.	0.73	0.901	0.66	4.74 ± 1.01	0.80	0.953	0.73	2.41 ± 1.18
	25	Measure whether the decision is satisfactory.	0.79	0.916	0.65	4.29 ± 1.04	0.77	0.964	0.65	2.80 ± 1.24
	26	Adapt decisions to the preferences of each patient.	0.62	0.910	0.52	4.36 ± 1.10	0.73	0.938	0.65	2.65 ± 1.25
**D2** Using information to see the big picture			α = 0.817 λ2 = 0.820				α = 0.885 λ2 = 0.887			
	1	Identify patterns	0.75	0.905	0.62	3.98 ± 1.02	0.75	0.914	0.68	3.11 ± 1.21
	2	Relate relevant clinical information to the problem.	0.76	0.895	0.68	3.91 ± 1.00	0.84	0.881	0.77	3.21 ± 1.23
	3	Assess the clinical picture in general.	0.76	0.883	0.67	3.59 ± 1.12	0.83	0.903	0.76	3.36 ± 1.26
	4	Remember past knowledge and apply it.	0.60	0.922	0.47	4.13 ± 1.05	0.76	0.950	0.62	3.04 ± 1.26
	6	Identify findings and relate them to the problem.	0.73	0.919	0.62	3.80 ± 1.03	0.79	0.947	0.71	3.26 ± 1.11
	7	My decision improves laboratory parameters.	0.70	0.919	0.55	3.86 ± 1.18	0.77	0.940	0.64	3.19 ± 1.29
	13	Anatomy and physiology to evaluate.	0.66	0.931	0.42	3.77 ± 1.55	0.71	0.956	0.56	3.32 ± 1.26
**D3** Knowing and acting			α = 0.795 λ2 = 0.811				α = 0.839 λ2 = 0.857			
	5	Implement the best option.	0.23	0.801	0.22	3.43 ± 0.74	0.23	0.790	0.11	3.35 ± 0.88
	14	Use intuition to act.	0.66	0.916	0.57	3.79 ± 1.15	0.78	0.941	0.73	3.52 ± 1.31
	15	Analyze the risks.	0.71	0.943	0.49	4.00 ± 0.99	0.81	0.963	0.68	3.28 ± 1.24
	17	Independently decide the solution.	0.76	0.908	0.68	3.58 ± 1.28	0.84	0.934	0.76	3.74 ± 1.40
	20	Act on an urgent problem.	0.73	0.930	0.65	3.65 ± 1.29	0.72	0.932	0.65	3.73 ± 1.45
	21	Use knowledge in complementary tests to decide.	0.66	0.929	0.54	3.57 ± 1.29	0.72	0.960	0.58	3.37 ± 1.38
	27	Consider interventions that only “seem” correct.	0.54	0.901	0.49	3.50 ± 1.21	0.67	0.950	0.58	3.43 ± 1.29

I = Items, Load factor estimated CFA, KMO = Kaiser-Meyer-Olkin, Item *r* = correlation item– sub dimension, M ± SD = Media and Standard Deviation, α = Cronbach’s alfa coefficients, λ2 = Guttman split-half coefficients (lambda-2 statistic). The short descriptions of the items are not the full verbiage for the NASC-CDM© scale

*Explanatory note: To use the original version, the Spanish version NASC-CDM-S© or in any of the translated and validated version in other languages, you must contact the original author and copyright holder of the scale: Dr. Krista White. krista.white@georgetown.edu or kawhite4288@gmail.com

Highly significant correlations were found regarding criterion validity and relevance (*p* <.001). Correlations within the dimensions within the same scale (D1, D2, D3) were positive, whereas the paired correlations between self-confidence and anxiety were inversely correlated, as increased confidence was associated with decreased anxiety: (D1 *r* = −.500), (D2 *r* = −.500) and (D3 *r* = −.532).

### Comparative analysis of self-confidence and anxiety in decision making by academic year

The overall results for self-confidence and anxiety by academic year indicated that students significantly and gradually increased their self-confidence (*p* =.049) as they progressed from the 1st year (M = 108.22, SD = 14.96) to the 4th year (M = 115.54, SD = 16.28). However, anxiety was higher in the 1st year (M = 81.71, SD = 18.90) and increased in the 3rd year (M = 86.32, SD = 26.38) (Table 3).

**Table 3:** Overall self-confidence/ anxiety global and academic year (1st, 2nd, 3rd, 4th)

			Self-confidence						Anxiety				
Year	*n*		Mean	SD	F	*p*	η²		Mean	SD	F	*p*	η²
1st	86		108.22	14.96	2.647	0.049	0.026		81.71	18.60	2.333	0.074	0.023
2nd	116		110.49	18.57					80.22	21.90			
3rd	40		114.15	16.90					86.32	26.38			
4th	59		115.54	16.28					75.05	21.14			

SD = Standard deviation, F, p, η² eta-squared effect size

Table 4 shows statistically significant differences in dimensions D2 and D3 for self-confidence and D3 for anxiety.

**Table 4:** ANOVA: Dimensions and academic year (1st, 2nd, 3rd, 4th)

Dimensions	Mean Square	F	*P*	η²
D1 - Self confidence	39.95	0.484	0.694	0.005
D1 - Anxiety	307.29	1.667	0.178	0.023
D2 - Self confidence	166.47	5.667	**< 0.001**	0.054
D2– Anxiety	106.54	2.447	0.064	0.024
D3 - Self confidence	168.54	6.085	**< 0.001**	0.058
D3– Anxiety	136.62	3.386	**0.019**	0.033

*Cases fixed factor Academic year, df1 (3), df2 (297), F, p, η² eta-squared effect size

#### Dimension D1 - using resources to collect information and listening carefully

**Fig. 1:**
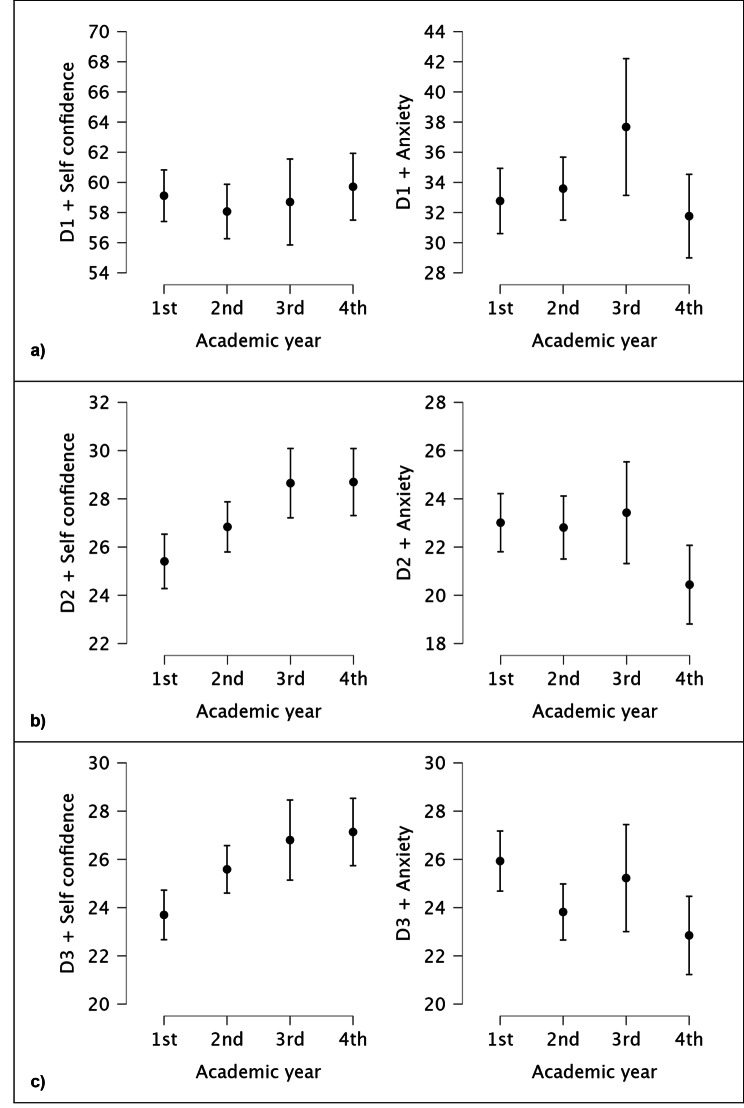
Comparison graphics of different dimensions of different Academic years (**a**) D1. Using resources to collect information and listening carefully: Post Hoc Comparisons Academic year (1st, 2nd, 3rd, 4th) (**b**) D2. Using information to see the big picture: Post Hoc Comparisons Academic year (1st, 2nd, 3rd, 4th). (**c**) D3. Knowing and acting: Post Hoc Comparisons Academic year (1st, 2nd, 3rd, 4th)

The post hoc Tukey test results indicate no statistically significant differences between academic years in dimension D1 (Table 4). Students in higher academic years did not obtain significantly higher self-confidence or lower anxiety scores (Fig. 1a). The self-confidence means were similar across all 4 groups, while the anxiety mean had varying values. The highest anxiety was observed in the 3rd year (M = 37.67; SD = 14.63), and the lowest was in the 4th year (M = 31.76; SD = 10.82), although the differences were not statistically significant (*p* =.178).

#### Dimension D2 - using information to see the big picture

Students in the higher academic years (3rd and 4th) obtained significantly higher self-confidence scores (M = 28.69; SD = 5.44) compared to the lowest, which is from the 1st year (M = 25.40; SD = 5.33) (Table 4; Fig. 1b). There was a downward trend in anxiety in the later years, but it was not significant. Once again, the highest mean anxiety was observed in the 3rd year (M = 23.42; SD = 6.80) and the lowest in the 4th year (M = 20.44; DS = 6.39).

#### Dimension D3 - knowing and acting

This is the only dimension where a balance was maintained: self-confidence increased with academic years, while anxiety decreased. Significant differences in self-confidence scores were observed between the 1st year (M = 23.70; SD = 4.85) and the 4th year (M = 27.13; SD = 5.47). At the same time, anxiety significantly decreased between the 1st year (M = 25.93; SD = 5.90) and the 4th year (M = 22.85; SD = 6.36) (Table 4; Fig. 1c).

### Effect of students’ work experience on their decision-making processes

A comparative test was conducted between groups based on work experience to identify explanatory variables regarding the extent of self-confidence and anxiety (Table 5). Two significant differences were found, indicating that students with work experience, as opposed to students without experience, had higher self-confidence in D2 (M = 27.66, SD = 5.43 vs. M = 26.63, SD = 5.61) and D3 (M = 26.24, SD = 5.52 vs. M = 24.58, SD = 5.10). Meanwhile, the level of anxiety was similar in both groups.

**Table 5:** Independent T-Test. Work experience (yes = 169, no = 132) by self-confidence/anxiety dimensions

	T	Df	*P*	Cohen’s d	SE Cohen’s d
D1 - Self confidence	0.271	299	0.787	0.031	0.116
D2 - Self confidence	2.244	299	**0.026**	0.261	0.117
D3 - Self confidence	2.665	299	**0.008**	0.310	0.117
D1– Anxiety	0.211	299	0.833	0.025	0.116
D2– Anxiety	-1.033	299	0.302	− 0.12	0.116
D3– Anxiety	-1.389	299	0.166	− 0.161	0.116

Student’s t-test, Cohen’s effect size

Furthermore, when contrasting individual items, 7 specific items showed significant differences in self-confidence and 2 in anxiety based on students’ work experience (Table 6).

**Table 6:** Comparison of self-confidence and anxiety with and without experience across items

Item	Self-confidence					Anxiety			
	t	*p*	Experience M ± SD	No experience M ± SD		t	*p*	Experience M ± SD	No experience M ± SD
I1	2.298	**0.022**	4.10 ± 0.98	3.83 ± 1.04		-2.378	**0.018**	2.96 ± 1.15	3.30 ± 1.26
I5	2.707	**0.007**	3.53 ± 0.71	3.30 ± 0.77					
I7	2.465	**0.014**	4.00 ± 1.12	3.67 ± 1.22					
I14	2.695	**0.007**	3.95 ± 1.14	3.59 ± 1.13		-2.056	**0.041**	3.38 ± 1.28	3.69 ± 1.33
I15	2.132	**0.034**	4.10 ± 0.98	3.86 ± 1.00					
I17	1.988	**0.048**	3.71 ± 1.25	3.42 ± 1.30					
I20	2.131	**0.034**	3.79 ± 1.32	3.47 ± 1.23					

Student’s t-test, M = Median, SD = Standard Deviation

Two items belong to D2- Using information to see the big picture, where experienced students exhibited greater self-confidence in detecting important patient information patterns in I1 (M = 4.10 vs. M = 3.98) and experienced less anxiety (M = 2.96 vs. M = 3.30), and simultaneously evaluated their decisions better with patient laboratory results in I7 (M = 4.00 vs. M = 3.67).

The other five items correspond to D3- Knowing and acting, where nursing students with prior nursing experience felt more self-confidence when deciding the best priority alternative for the patient’s problem in I5 (M = 3.53 vs. M = 3.30), more confidence in implementing an intuition-based intervention in I14 (M = 3.95 vs. M = 3.59) with less anxiety (M = 3.38 vs. M = 3.69), more confidence in analyzing the risks associated with interventions I15 (M = 4.10 vs. M = 3.86) a better ability to make autonomous clinical decisions in I17 (M = 3.71 vs. M = 3.42), and to implement a specific intervention in an emergency in I20 (M = 3.79 vs. M = 3.47).

## Discussion

Given the objectives and results of this study, the discussion is subdivided into two sections: (1) Study of the Nursing Anxiety and Self-Confidence with Clinical Decision Making (NASC-CDM©) scale from English to Spanish, and (2) Assessment of self-confidence and anxiety in nursing students.

### Study of the nursing anxiety and self-confidence with clinical decision making (NASC-CDM©) tool

The findings of this study highlight the successful adaptation and validation of the NASC-CDM© scale, originally developed by White [14, 21], into Spanish (NASC-CDM-S©). This adaptation process demonstrated high reliability in both self-confidence and anxiety scales. The psychometric study conducted confirmed the validity of the three original dimensions. This result was achieved by examining item concordance with the dimensions of the original scale, followed by CFA of the entire scale. This resulted in a total variance exceeding 40% for both scales and across dimensions, confirming construct validity. The Spanish version effectively maintains the three- dimension groupings (D1, D2 and D3), which also preserves the item descriptions. Consequently, the obtained results align closely with White’s original study [14] and the Turkish version [24]. Regarding the loading factor, only one item, I5, “Make a decision on the ‘best’ prioritized alternative for the user’s problem,” had a loading value below 0.30 [32]. While its factor loading was 0.23 and exhibited a low correlation with the other items (*r* =.22), its KMO ratio was ≥ 0.80, suggesting potential influence by underlying factors such as age or work experience. Therefore, the decision was made to retain it. However, these findings were not replicated in the translation of the NASC-CDM into Korean (KNASC-CDM) (KNASC-CDM) [22]. The Korean version comprises 23 items grouped into 4 groupings: (i) Listening fully and using resources to gather information; (ii) Using information to see the big picture; (iii) Knowing and acting; and (iv) Seeking information from clinical instructors.

The observed correlations between the dimensions of self-confidence and anxiety provide valuable and interesting insight. The results indicate an inverse relationship between the two, suggesting that strengthening self-confidence can have a positive impact on reducing anxiety. This aspect was corroborated by the original study by White [21] and Bektas et al. [13], demonstrating that metacognitive awareness increases nursing students’ self-confidence in clinical decision-making and reduces anxiety.

Furthermore, it is worth noting that the NASC-CDM© scale has been employed in numerous research studies related to nursing education. Therefore, its potential for educational purposes in both academic and clinical settings as a scale for measuring the enhancement of clinical decision-making skills is acknowledged. Several studies [33–35] suggest the effectiveness of in-person or virtual simulation in enhancing skills related to self-confidence in clinical decision-making, situational awareness, and communication effectiveness among students. Comparing the outcomes of this study with others utilizing the NASC-CDM© scale to gauge self-confidence and anxiety [33, 36], it was noted that self-confidence levels increase with diverse teaching strategies, while anxiety levels are not negatively impacted. Overall, these findings underscore the importance of the NASC-CDM© scale in assessing students’ readiness for decision-making, highlighting the necessity to address emotional factors such as anxiety and the need to bolster self-confidence to enhance the education and preparation of future nursing professionals for challenging clinical scenarios.

### Assessment of self-confidence and anxiety in nursing students

The results of the comparative study among nursing students across different academic years reveal an intriguing dynamic between self-confidence and anxiety throughout their academic progression. While self-confidence increases as students advance through their courses due to the acquisition of knowledge and skills, anxiety shows variations over time. Regarding confidence perception, some authors [37] claim that confident students learn better and that this self-confidence increases with experience, leading to improved knowledge [13].

One factor that might explain the difference in anxiety levels is that in the initial academic years (first and second), clinical practices are conducted in a more guided and supervised manner. In the third, and especially in the fourth year, clinical practices increase in terms of hours and complexity, requiring students to take on more responsibility and autonomy. This factor might account for the higher levels of anxiety in the third year, when students begin to engage in more autonomous practices and specialized units [38, 39]. This stage could induce anxiety due to the increased responsibility and potential consequences in patient care. In other words, even though students become more secure in their skills, they may also experience anxiety due to the weight of their clinical practice decisions in the knowledge that they will soon be certified professional nurses caring for patients. This duality is understandable in a context where decision-making has direct implications for patient health and the potential consequences of their actions in patient care. However, this situation is rectified in the fourth or final year, when anxiety decreases, and self-confidence increases. Clinical experience helps students develop skills and self-confidence, which, in turn, reduces anxiety [15, 40]. Just as in the case of nurses, the benefits of experience in decision-making are evident in students [3]. However, some researchers [41] emphasize the need to reinforce training in aspects such as situational awareness and cognitive apprenticeship to develop decision-making skills in senior students. There is evidence linking emotion and cognition to clinical decision-making [42].

Results from this study allow for a more detailed analysis by dimensions (D1, D2, D3) across academic years. Dimension 1 - *Using resources to gather information and listening fully* (D1) is the only dimension that does not show significant differences by year in either self-confidence or anxiety. This dimension includes fundamental aspects of assessment and information gathering (verbal and non-verbal communication, the ability to review the literature, and information provided by others, among others) [14]. In Dimension 2 - *Using information to see the big picture* (D2), self-confidence significantly increases, and anxiety decreases, although the latter is not statistically significant. This dimension encompasses aspects related to interpreting information to identify the patient’s actual problem, filtering out irrelevant information, and applying knowledge to the detected problem [14]. Finally, Dimension 2 - Knowing *and acting* (D3) - is the only dimension that behaves as hypothesized, with increasing self-confidence and decreasing anxiety. This dimension includes aspects related to training in addressing the problem and detecting the repercussions of the interventions performed, as well as the student’s autonomous ability to address the detected problem [14].

The results indicate that although students demonstrate skills in applying knowledge and performing interventions (D2 and D3), there appears to be a lack of training proficiency in the comprehensive assessment of the patient as an individual with specific needs (D1). This shortcoming is likely caused by various factors, including lack of experience, inadequate training skills, and the complexity of the assessment process. Understanding the patient is a complex task, as nurses must consider not only physiological indicators. Therefore, this requires time and experience [3] This implies that students tend to focus more on pathology and standardized care rather than on the patient as a unique individual with specific needs and characteristics.

In contrast, in the case of nurses, when patients do not align with their prior experience, nurses are more motivated to assess the patient and facilitate decision making [3]. The need for a proper and personalized patient assessment emerges as a crucial point for improvement in the education of nursing students [43]. Therefore, an educational intervention focused on strengthening the skill of patient assessment throughout the nursing degree program could favor the development of nursing students as future professionals. Such an intervention could include the implementation of more effective assessment tools and the promotion of careful observation of all aspects of the patient. It should extend beyond nursing-specific procedures involving the development of cognitive skills [44]. Importantly, it should be implemented not only in the academic context but also in the clinical setting. Given that education alone is not an ideal measure [3], this clinical involvement is essential based on patient-centered health care ( [45].

Finally, in relation to students with work experience, those who work as nursing assistants during their nursing education exhibit more self-confidence and less anxiety in various items: seeing patterns in patient information (I1) and implementing interventions based on gut feeling or intuition (I14). They also demonstrate higher self-confidence when making a decision about the ‘best’ priority decision option for the patient’s problem (I5), evaluating whether their clinical decision improved the patient’s laboratory results (I7), analyzing the risks of the interventions (I15), making independent clinical decisions to solve the patient’s problem (I17), and implementing a specific intervention in case of an urgent problem (I20). It can be affirmed that experienced students show more self-confidence in having a holistic view of the patient (D2) and in their knowledge and patient-related actions (D3). Other studies [46] detail the benefits of work experience in emotional control and stress reduction among students. Moreover, students’ prior work experience contributes to decision making, as it provides them with a more realistic understanding of the role and responsibilities of the nursing profession [47].

### Limitations

Due to its cross-sectional design, this study prevents the establishment of causal relationships between self-confidence and anxiety. The study sample was limited to a specific group of students from a single Spanish-speaking university. Similar to the study by Bektas [24] only voluntary students participated in this study. It is pertinent to acknowledge potential biases in interpreting differences by academic year, as the sample is disproportional in one of the strata (with 9% margin of error), attributed to the absence of third-year students engaged in mobility programs and clinical practices. Moreover, the present study did not evaluate organizational and nursing practice factors, which could explore nursing students’ perceptions regarding clinical decision-making. Finally, even though the availability of the SNASC-CDM will facilitate its use in other Spanish-speaking countries, it is advisable to conduct specific studies to ensure its validity in a cultural context different from Spain.

## Implications for nursing education

Nursing degree programs should prioritize the development of students’ self-confidence and the management of their anxiety. This could involve implementing educational interventions, including clinical simulation and reflective teaching that incorporate elements of metacognition. Collaboration across different subjects is essential to foster the integration of skills and knowledge. It is also vital that nursing programs provide students with opportunities to develop their clinical and communication skills. This will help students feel more secure in their abilities and reduce anxiety in challenging clinical settings.

The findings of this study suggest that nursing students face challenges in assessing patients, which can be attributed to various factors, including lack of time, insufficient training, and limited experience. To address this issue, an educational intervention is proposed for nursing students. This intervention would focus on conducting a comprehensive and holistic patient assessment with the support of practicing nurses and involving the patients themselves in identifying problems and needs. Such an intervention should include discussing the significance of considering the patient’s physical, emotional, spiritual, and social needs. It should also emphasize the importance of building a trusting relationship with the patient.

## Conclusions

The Spanish version of the NASC-CDM (NASC-CDM-S©) allows for the identification of self-confidence and anxiety in clinical decision-making in Spanish-speaking nursing students. Moreover, it retains the same structure as the original English version. The availability of the NASC-CDM-S© will facilitate its use in other Spanish-speaking countries, thus enhancing the education and preparation of future nursing professionals in clinical situations.

Self-confidence increases as students progress through their academic years due to knowledge and skills acquisition, while anxiety shows variations over time. Specifically, anxiety tends to increase in the third year, when students transition to more autonomous practices and specialized health care units. However, diverse perceptions are identified depending on the dimension. The only dimension that achieves a positive balance in self-confidence and anxiety is D3 (Knowing and acting). Nevertheless, the findings reveal deficiencies in D1 (*Using resources to gather information and listening fully)* regarding assessing and detecting problems.

Students with prior work experience show improved self-confidence in D2 and D3, but the level of anxiety does not differ between students with and without work experience. Therefore, targeted interventions addressing emotional and cognitive aspects are needed to enhance clinical decision-making and provide better patient care. Considering these aspects, future lines of research could explore the impact of teaching interventions, as well as conduct further studies on the NASC-CDM-S©, validating it in different Spanish-speaking countries, and applying it in clinical settings with healthcare professionals.

## Data Availability

No datasets were generated or analysed during the current study.
